# Whole Genome Sequencing in Cancer Clinics

**DOI:** 10.1016/j.ebiom.2014.12.007

**Published:** 2014-12-18

**Authors:** Ken Chen, Funda Meric-Bernstam

**Affiliations:** a Department of Bioinformatics and Computational Biology, The University of Texas MD Anderson cancer center, United States; b Department of Investigational Cancer Therapy, The University of Texas MD Anderson cancer center, United States; c Institute of Personalized Cancer Therapy, The University of Texas MD Anderson cancer center, United States

Patients with a family history of cancer are being evaluated with single-gene or gene panel tests (LaDuca et al., 2014). The decreasing cost and potential to provide comprehensive genetic risk assessment makes whole genome sequencing (WGS) an attractive tool for understanding the genetic risk for cancer (Collins and Hamburg, 2013). Despite such potential, the empirical value of WGS in cancer genetics clinics is unknown: whether WGS can replicate previous findings in single/multi-gene testing, and whether it will increase the rate of identification.

In this issue of *EBioMedicine*, Foley et al. describes their results obtained from analyzing the WGS data from the blood samples of two cohorts of cancer genetics patients: those with *BRCA*1/2 mutations (n = 176) and those without (n = 82) (Foley et al., 2015-in this issue). In their analysis, they focus on a set of 163 clinically relevant genes, including those on commercial cancer-susceptibility gene panels, recommended by American College of Medical Genetics (Green et al., 2013), and those that might impact reproductive decision-making (Dorschner et al., 2013). They find that in each *BRCA*1/2 patient there is an average of 6.8–6.9 potentially pathogenic variants (PPVs), defined as nonsynonymous variants with allele frequency < 1% in a normal human population (ESP6500). All the previously known *BRCA*1/2 mutations are detected, proving the sensitivity of WGS.

As anticipated, most of these PPVs are missense variants, the majority of which will be classified as variants of unknown significance (VUS) based on existing knowledge (Biesecker, 2012). To facilitate diagnoses, Foley et al. further restrict analyses to only loss-of-function (LoF) PPVs, including nonsense, nonstop single nucleotide variants (SNVs) and frame-shift indels (Foley et al., 2015-in this issue). In six patients, they identify LoF PPVs in four dominant cancer-associated genes (*CHEK2*, *ATM*, *RAD50*, and *CDKN2B*), in addition to original clinically diagnosed *BRCA*1/2 mutations. These variants that carry additional cancer risk may be of use for counseling and then screening of the patient's family members.

Interestingly, they find that previously reported pathogenic missense variants in several genes do not associate with their predicted diseases. For example, the missense variant *CREBBP* p.N1978S is previously reported as pathogenic and diagnostic for Rubenstein Taybi syndrome (RTS) (Roelfsema and Peters, 2007). However, a patient carrying this variant and her family members that carried the variant have no symptoms consistent with a RTS diagnosis. The missense variant *PRSS1* p.N29I is previously associated with hereditary pancreatitis and subsequent pancreatic cancer. None of nine patients that carried the *PRSS1*-variant report any pancreatic problems. These results indicate that while WGS can expand the breadth of disease risk analysis, clinical interpretation of WGS results, particularly for missense variants, requires detailed medical histories and results from extended family members to derive confident diagnoses for appropriate genetic counseling. Moreover, previously reported disease associations should be understood with caution and be interpreted in light of available family medical history.

Among the 82 unrelated non-*BRCA* patients, WGS identifies 6.4 PPVs per non-patient in the 163 genes, including LoF PPVs in 14 genes in 18 (22%) patients. Notably, of the 13 individuals with LoF PPVs in cancer-associated genes, 11 are in genes implicated in the individual's primary cancer diagnosis and two provided a likely genetic diagnosis based on family history. WGS also detects LoF PPVs in cancer genes such as *PALB2* and *RAD51C*, which are beyond tests performed as standard of care for this population. This again illustrates the potential of WGS to identify co-occurring mutations that pose genetic risks beyond the patient's primary diagnosis or that may be modifiers of cancer risk.

To increase the diagnosis rate to the non-*BRCA* patients, the authors expand analysis to include genes annotated in the ClinVar database (Landrum et al., 2014). They identify LoF SNV PPVs in previously unsuspected cancer genes that are not considered as part of standard clinical care, including *ERCC3*, *FANCA* and *FANCM*, which are good candidates for further research.

In total, WGS provides possible genetic cancer risk PPVs in 20.7% of non-*BRCA*1/2 clinic patients, doubling the rate (10.6%) of a recent report using a targeted 42 gene panel of non-*BRCA*1/2 patients (Kurian et al., 2014). It is clear that WGS can enhance discovery of additional PPVs. However, it should be emphasized that at this time for many of these additional genes, adequate natural history data is lacking, making appropriate genetic counseling, risk assessment, and recommendations for future screening and preventive strategies very challenging. Thus significant research efforts in larger patient cohorts will be needed before clinical WGS is widely adopted. The value of WGS will further increase as clinical evidence becomes available for additional variants, particularly copy number variants and variants in non-coding regions.

Overall, this study highlights the ongoing discussion as to the appropriateness and use of WGS to balance the research goals to improve future health care with the goals of improving current patients' health and well-being. It also indicates that increasing the clinical use of WGS will require large-scale efforts to consolidate WGS results with clinical data to improve accuracy of interpretation of rare variants, and to determine optimum management strategies.

## Disclosure

The authors declared no conflicts of interest.

## References

[bb0030] Biesecker L.G. (2012). Opportunities and challenges for the integration of massively parallel genomic sequencing into clinical practice: lessons from the ClinSeq project. Genet. Med..

[bb0010] Collins F.S., Hamburg M.A. (2013). First FDA authorization for next-generation sequencer. N. Engl. J. Med..

[bb0025] Dorschner M.O., Amendola L.M., Turner E.H., Robertson P.D., Shirts B.H., Gallego C.J. (2013). Actionable, pathogenic incidental findings in 1,000 participants' exomes. Am. J. Hum. Genet..

[bb0015] Foley S.B., Rios J.J., Mgbemena V. (2015). Use of whole genome sequencing for diagnosis and discovery in the cancer genetics clinic. EBioMedicine.

[bb0020] Green R.C., Berg J.S., Grody W.W., Kalia S.S., Korf B.R., Martin C.L. (2013). ACMG recommendations for reporting of incidental findings in clinical exome and genome sequencing. Genet. Med..

[bb0045] Kurian A.W., Hare E.E., Mills M.A., Kingham K.E., McPherson L., Whittemore A.S. (2014). Clinical evaluation of a multiple-gene sequencing panel for hereditary cancer risk assessment. J. Clin. Oncol..

[bb0005] LaDuca H., Stuenkel A.J., Dolinsky J.S., Keiles S., Tandy S., Pesaran T. (2014). Utilization of multigene panels in hereditary cancer predisposition testing: analysis of more than 2,000 patients. Genet. Med..

[bb0040] Landrum M.J., Lee J.M., Riley G.R., Jang W., Rubinstein W.S., Church D.M., Maglott D.R. (2014). Clinvar: public archive of relationships among sequence variation and human phenotype. Nucleic Acids Res..

[bb0035] Roelfsema J.H., Peters D.J. (2007). Rubinstein–Taybi syndrome: clinical and molecular overview. Expert Rev. Mol. Med..

